# Molecular phylogeny and morphometric divergence of native Korean wild mice (*Mus musculus*)

**DOI:** 10.1186/s42826-026-00269-7

**Published:** 2026-03-25

**Authors:** Daewoo Kim, Jooseong Oh, Jang Geun Oh, Hee-Young Yang, Geun-Joong Kim, Tae-Hoon Lee, Bae-Keun Lee, Chungoo Park, Dong-Ha Nam

**Affiliations:** 1 Department of Biological Sciences and Institute of Sustainable Ecological Environment, College of Natural Sciences, Chonnam National University, Gwangju, 61186 Republic of Korea; 2 School of Biological Sciences and Technology, College of Natural Sciences, Chonnam National University, Gwangju, 61186 Republic of Korea; 3 Jeju Wildlife Problem Institute, Jeju-do, 63089 Republic of Korea; 4 Preclinical Research Center, Daegu-Gyeongbuk Medical Innovation Foundation (K-MEDIhub), Daegu, 41061 Republic of Korea; 5 Department of Oral Biochemistry, Dental Science Research Institute, School of Dentistry, Korea Mouse Phenotype Center (KMPC), Chonnam National University, Gwangju, 61186 Republic of Korea; 6 National Institute of Ecology, Seocheon-gun, Chungcheongnam-do 33657 Republic of Korea

**Keywords:** House mouse, Taxonomy, Phylogeny, Morphology, Korean Peninsula

## Abstract

**Background:**

The taxonomic status of house mice (*Mus musculus*) on the Korean Peninsula has long been debated due to conflicting morphological classifications and limited genetic evidence. Historically, three subspecies (*M. m. molossinus*, *M. m. utsuryonis*, and *M. m. yamashinai*) have been proposed based on external traits, although the validity of these proposals remains uncertain. Thus, this study aimed to integrate genetic and morphological analyses to clarify the phylogenetic relationships of Korean mice relative to the well-known primary *M. musculus* subspecies and evaluate the taxonomic distinctiveness.

**Results:**

Genetic analysis of mitochondrial DNA (*cytb* gene) from mice across Korea, including islands, mountains, and agricultural fields, confirmed that these mice belong to the Eurasian *M. m. musculus* lineage. Morphologically, Korean mice exhibited tail ratios consistent with previously assigned subspecies, suggesting these traits represent intraspecific variation within *M. m. musculus*. Craniometric analyses revealed distinctive features, such as a shorter, narrower premaxillary tooth-patch width and a longer maxillary tooth-row length, thereby distinguishing these mice from laboratory strains derived from *M. m. domesticus*. These cranial configurations, visualized via three-dimensional micro-computed tomography scans, further supported the morphological divergence of these mice from other subspecies.

**Conclusions:**

Our findings indicate that Korean house mice belong to a single subspecific group within *M. m. musculus*, with observed morphological variations reflecting local adaptation rather than distinct taxonomic divisions. The Korean Peninsula likely served as an ecological bridge, facilitating the spatiotemporal diversification of *M. m. musculus* across East Eurasia. This study resolves longstanding taxonomic ambiguities and underscores the subspecific status of Korean house mice within *M. m. musculus*. These insights provide a foundation for understanding the biogeographic history of human commensal species and future biomedical research utilizing wild-derived mouse models.

**Supplementary Information:**

The online version contains supplementary material available at 10.1186/s42826-026-00269-7.

## Background

Wild house mice (*Mus musculus*) are human commensals whose ranges have expanded alongside human activities, facilitating the continuous accumulation of morphological and genetic variations. The *Mus musculus* species originated in the Indian subcontinent and has since dispersed worldwide, diverging into three primary subspecies: *M. m. castaneus* (CAS), which are present throughout Southeast Asia; *M. m. domesticus* (DOM), distributed from Western Europe and Africa to North and South America; *M. m. musculus* (MUS), whose range has expanded throughout Eurasia [1].

The Korean Peninsula in the Far East of Eurasia presents ecological barriers that influence the distribution and population dynamics of mammals. Indeed, based on external body measurements and fur or hair color phenotypes, previous studies have classified wild mice on the Korean Peninsula into three subspecies: *M. m. molossinus* [2, 3], *M. m. utsuryonis* [4], and *M. m. yamashinai* [5]. However, classifying species based on subtle morphological variations remains controversial, as such classifications may not accurately reflect the true evolutionary relationships (phylogeny) of these mice. Given that MUS in East Eurasia may have entered the Korean Peninsula from northern China, with the geographical dispersal presumably facilitated by human immigration [6, 7], the question remains whether these assigned mouse subspecies truly reflect the correct taxonomic positions [8–14]. Despite existing genetic [6, 7, 11–14] and morphological [8–14] studies on Korean mice, to our knowledge, no previous research has simultaneously examined both aspects in Korea at the type localities of the three nominal subspecies (*M. m. molossinus* [2, 3], *M. m. utsuryonis* [4], and *M. m. yamashinai* [5]).

Therefore, this study aimed to integrate existing genetic and morphological data from house mice with mtDNA markers, three-dimensional (3D) micro-computed tomography (CT)-scanned landmark data, and craniometrics to address the evolutionary relationships of house mice in the Korean Peninsula. This approach can clarify the phylogenetic relationships of these mice for the three primary house mouse subspecies.

## Methods

### Samples

**Fig. 1:**
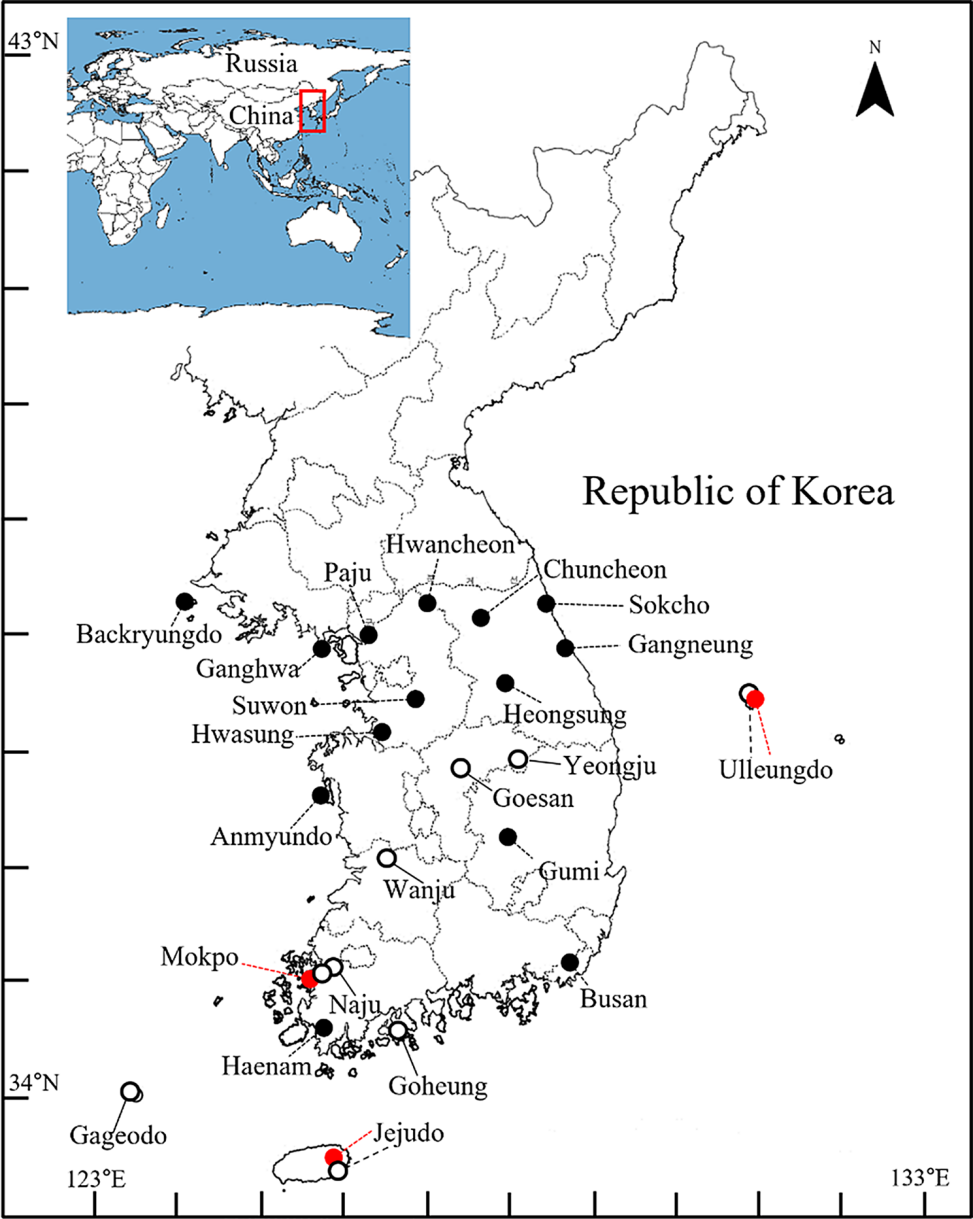
The Korean house mice sampling locations in the present study (open circle). The red circles (type localities) indicate the three subspecies that were assigned morphologically: *M. m. molossinus* on Jeju Island, *M. m. utsuryonis* on Ulleung Island, and *M. m. yamashinai* on the Korean Peninsula (including Mokpo) [2–5]; the solid black circles indicate previously studied locations for the Korean mice lineage (Additional file 1: Table S2)

Wild mice were collected from various locations across Korea, including islands (Gageo-do, Jeju-do, and Ulleung-do; *n* = 6), mountains (Yeongju-si and Wanju-gun; *n* = 3), and agricultural fields (Goesan-gun, Goheung-gun, and Naju-si; *n* = 14) between 2017 and 2023 (Fig. 1; Additional file 1: Table S1). These samples were collected from typical localities of the three nominal subspecies (*M. m. molossinus* [2, 3], *M. m. utsuryonis* [4], and *M. m. yamashinai* [5]) in Korea to ensure that increasingly robust molecular and morphometric evaluations were performed. In addition to these wild mice, we used four commercially available laboratory mouse strains (CBA, C57BL/6H, C3H, and BALB/c derived from DOM) and our inbred lines from Goesan-gun (KG01BRA/1CNUf1–f3 and KG01BRA/1CNUm1–m3); each group included three male and three female mice, aged 9 weeks. Sexual dimorphism in mice was determined by examining the development of the external genitalia. This animal study was approved by the Chonnam National University Institutional Animal Care and Use Committee (CNU IACUC-YB-2021-103). This study was performed in accordance with the regulations set by the Chonnam National University Institutional Animal Care and Use Committee.

### Molecular analysis

Surface contaminants were removed from the tail tips (approximately 0.5 cm in length) of 23 specimens by scraping the skin using a sterilized scalpel, from which genomic DNA was extracted. The mitochondrial *cytochrome b* gene (*cytb*) (1140 bp) was amplified using the polymerase chain reaction (PCR) with universal primers (L7, ACC AAT GAC ATG AAA AAT CAT CGT T; H6, TCT CCA TTT CTG GTT TAC AAG AC). The PCR amplification conditions for *cytb* were as follows: initial denaturation at 95 °C for 5 min, followed by 36 cycles of denaturation (95 °C for 40 s), annealing (56 °C for 60 s), and extension (72 °C for 60 s). A final extension step was performed at 72 °C for 5 min. The purified PCR products were sequenced using the BigDye v3.1 (ABI).

We retrieved publicly available *cytb* sequences for the *M. musculus* subspecies and representative sequences of a closely related species (e.g., *M. spicilegus*) as an outgroup. Our mtDNA sequence data for each region were deposited in GenBank under the accession numbers of OQ506519.1–OQ506540.1 and AO53266.1 (Additional file 1: Table S1). MEGA11 was used to align the sequence data from the wild population mice subspecies and wild-derived subspecies strains. The mouse population number was used for the analysis of molecular variance (AMOVA) and to calculate Nei’s genetic distance based on *p*-distance; these were determined using the MEGA11 program. From the AMOVA, we obtained the fixation index (*Fst*), which describes the processes leading to genetic differentiation among and within populations. Haplotype networks were constructed for 93 *cytb* sequences (Additional file 1: Table S1) using the median-joining network to illustrate potential clustering within the main network topology, as a reticulation split. Maximum likelihood (ML) and Bayesian inference (BI) phylogenies were constructed for each *cytb* dataset (Additional file 1: Table S1) using the optimized Hasegawa–Kishino–Yano substitution model based on MEGA 11 and BEAST v1.10.4, respectively. The Markov chain Monte Carlo model was performed for 1,000,000 iterations, and the trees were sampled every 1000th generation. Nodal robustness was assessed using the Bayesian posterior probability (PP), with a PP > 95% considered strong support from the BI analysis. The same model was used to conduct the ML tree, and the ML bootstrap (BS) analysis was performed with 1000 bootstrap replications. A subgroup was clustered within each of the major mtDNA lineages only if bootstrap values > 70% were considered strong support from the ML analysis, combined with concordant structure in the haplotype networks. To assess demographic history, the haplotype number (*H*), haplotype diversity (*Hd*), and nucleotide diversity (*π*) were evaluated using DnaSP software. Tajima’s *D* and Fu’s *Fs* values were also estimated using ARLEQUIN software.

### Morphometrics

The micro-CT scanning system was used to capture elaborate tomographic images of each specimen. The specific procedures used in this study have been described previously with some minor adjustments [15, 16]. Each mouse was placed on an acrylic plate attached to an animal bed inside the micro-CT scanner. The vertebral column was carefully stretched to ensure accurate measurements of bone length in the resulting images, and the head, body, and tail were gently pressed onto the plate. The bone structures of the specimens were examined using a micro-CT scanning system combined with a Quantum GX micro-CT imaging system (PerkinElmer). A 3D micro-CT scanning system equipped with an X-ray tube (voltage, 90 kV; current, 80 mA) was used to scan the skeleton of each mouse. For the whole-body scans, a voxel size of 90 μm, a field of view (FOV) of 45 mm, a working distance of 108 mm, and a scan time of 2 min were applied in high-resolution scan mode. Cranial images were acquired using a voxel size of 50 μm, FOV of 25 mm, working distance of 55 mm, and scan time of 2 min in standard scan mode. The scanned skeletal data were reconstructed into 3D tomograms comprising high-contrast images of the skeletal parts of interest.

**Fig. 2:**
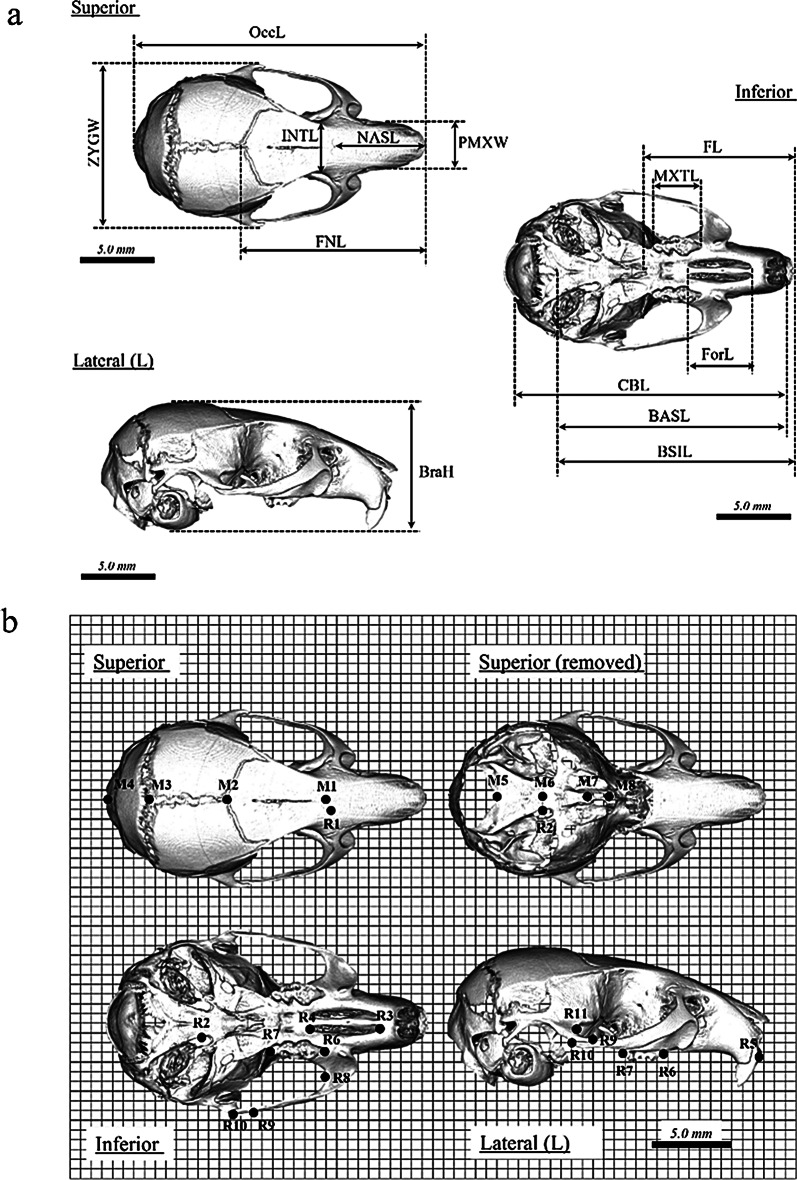
Linear cranial and dental variable measurements on micro-CT images (**a**). Abbreviations for the 14 cranial variables are as follows: OccL, occipitonasal length; ZYGW, maximum width of the zygomatic arch; INTL, interorbital constriction width; NASL, nasal length; PMXW, premaxillary width; FNL, frontonasal length; BraH, braincase height on tympanic bullae; FL, frontal bone length; MXTL, maxillary teeth row length; ForL, foramina incisive length; CBL, condylobasal length; BASL, basal length; BSIL, basilar length; NW: nasal bone width. Digitized landmarks (M1–8; R1–11) in the cranium (**b**): M1, posterior of nasal bone; M2, intersection of coronals suture and sagittal suture; M3, intersection of anterior lambdoid suture and sagittal suture; M4, caudal of skull; M5, lowest point of foramen magnum; M6, intersection of basisphenoid occipital bone; M7, intersection of presphenoid basisphenoid; M8, anterior of presphenoid; R1, intersection of premaxilla, nasal bone and front bone; R2, intersection of basisphenoid occipital bone; R3, rostral of rostral palatine foramen; R4, caudal of rostral palatine foramen; R5, frontal tip of incisors; R6, rostral tip of first molar; R7, caudal tip of third molar; R8, lowest point of zygomatic bone; R9, rostral point of zygomaticotemporal suture; R10, caudal of zygomaticotemporal suture; R11, posterior tip of zygomatic arch

To quantify both the size and shape of the geometric objects, we used the Micro-CT Viewer (Quantum GX, PerkinElmer) and ImageJ software. We measured the lengths of the following skeletal parts from wild mice (*n* = 13), four laboratory mouse strains (CBA, C57BL/6H, C3H, and BALB/c derived from DOM) (6 per strain), and our inbred mouse lines from Goesan-gun (*n* = 6) using Micro-CT and ImageJ: head and body (from the snout to the anus), tail (from the anus to the tip of tail), tail ratio (head and body length/tail length ⋅ 100) (Additional file 1: Fig. S1), and cranial linear measurements (nasal, frontal, parietal, interparietal, interparietal bone, maxillary and mandible bones) (Fig. 2). The craniometric variables were log-transformed to meet the assumption of normality. We compared 30 digitized landmarks in the cranium of our inbred mouse lines (*n* = 6; 9 weeks old) with four laboratory mouse strains (CBA, C57BL/6H, C3H, BALB/c) (6 per strain; 9 weeks old). This analysis aimed to depict the morphospace of the cranium in wild mice from Geosan-gun (Fig. 2). Position, orientation, and scaling biases were standardized using the generalized Procrustes analysis. Variations in skull shape and inter-subspecies distance between the mean shapes of each species were examined using the Geomorph package (v. 3.0.1) in R via a scatter plot of relative coordinates. All statistical analyses were conducted using R software (version 4.2.2).

## Results

### Phylogeny among Korean mice and the relationship to the *M. musculus* subspecies

**Fig. 3:**
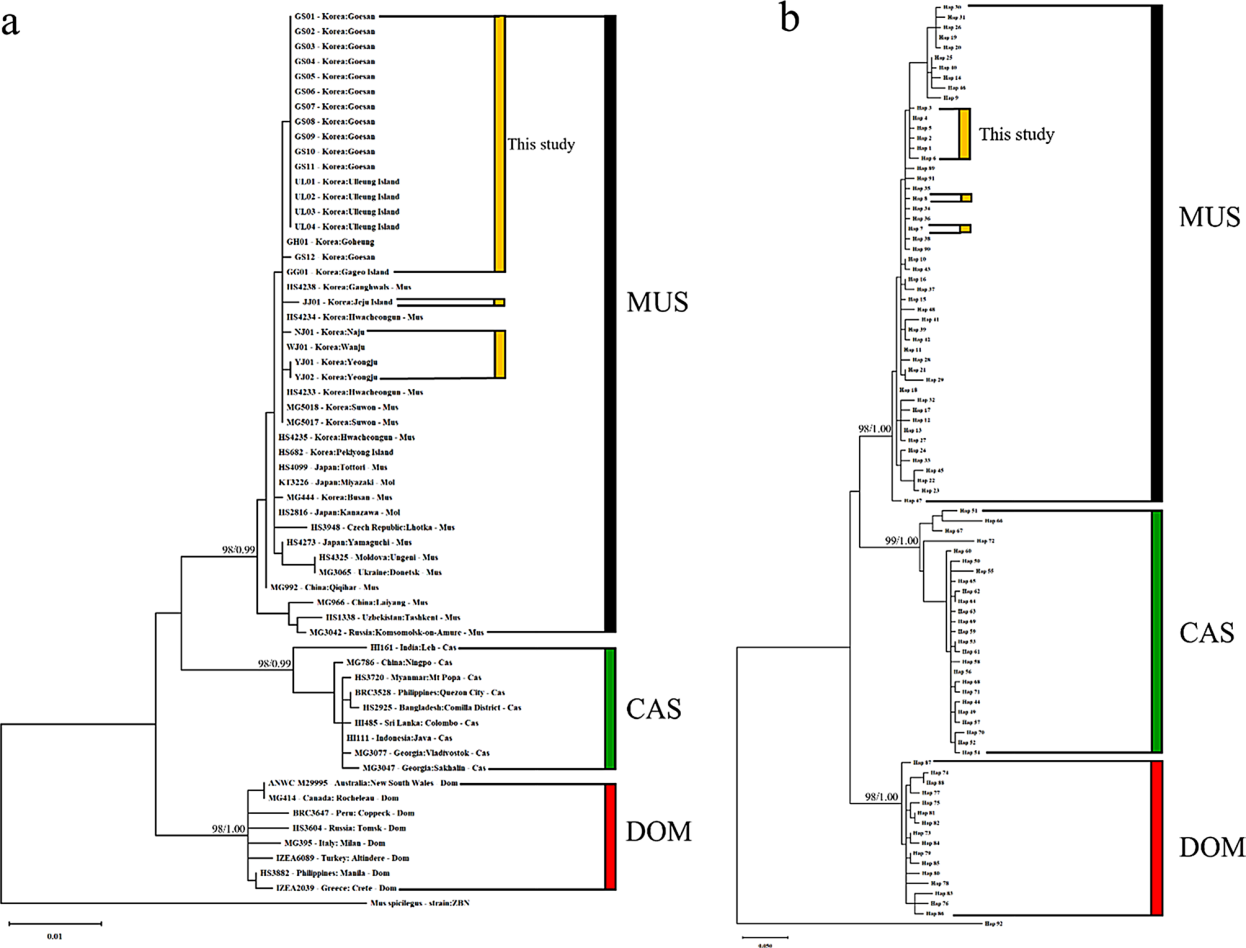
Maximum likelihood (ML) and Bayesian inference (BI) phylogenies based on mitochondrial *cytb* sequences in *Mus musculus* (**a**). The reconstructed ML/BI tree from haplotypes of mitochondrial *cytb* sequences in *Mus musculus* (**b**). The phylogroups represent the three subspecies groups: *M. m. musculus* (MUS), *M. m. castaneus* (CAS), and *M. m. domesticus* (DOM). The evolutionary history was inferred using the ML/BI method and the Hasegawa–Kishino–Yano model with the *cytb* datasets (Additional file 1: Table S1). The ML bootstrap/BI posterior probability (BS/PP) support values are indicated above the nodes. *Mus spicilegus* (strain: ZBN) is used as an outgroup. The lower left scale bar shows the number of nucleotide substitutions per site, representing the genetic distance between sequences in the phylogenetic tree

**Fig. 4:**
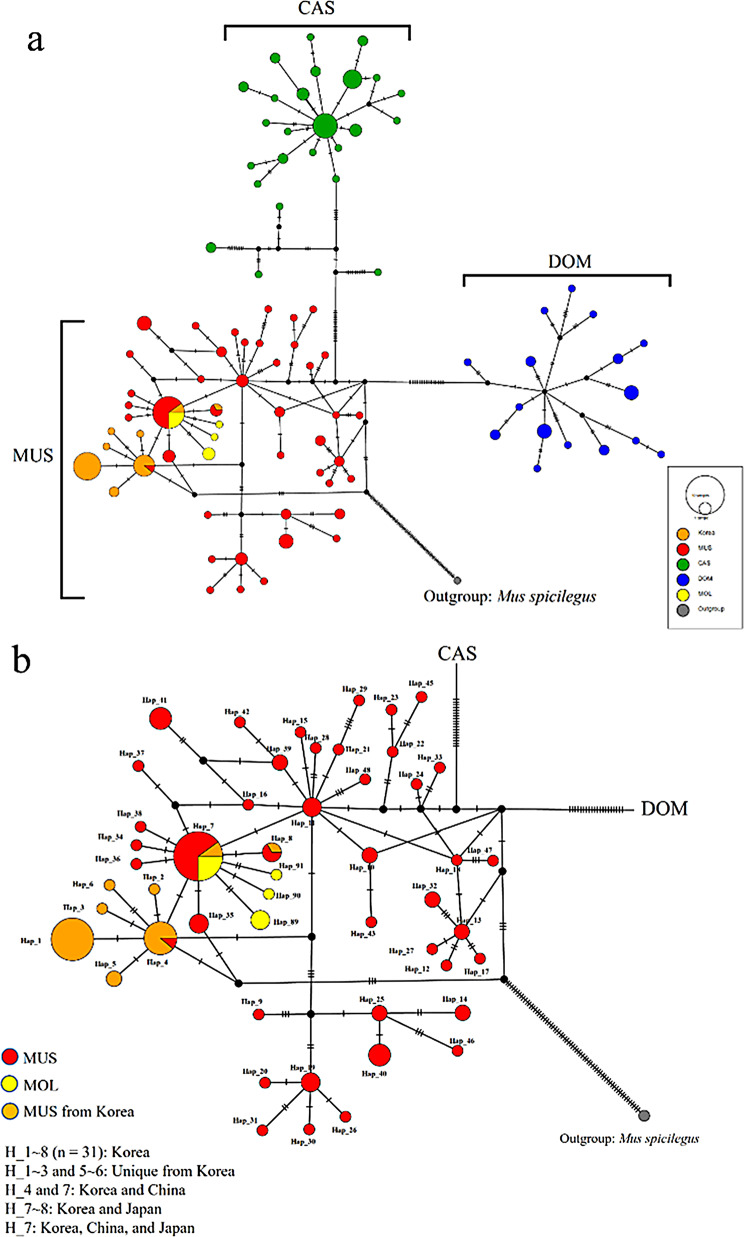
Haplotype network tree based on the mitochondrial *cytb* sequences in *Mus musculus* (**a**). The phylogroups represent the three subspecies groups: *M. m. musculus* (MUS), *M. m. castaneus* (CAS), and *M. m. domesticus* (DOM). We retrieved publicly available *cytb* sequences from the *M. musculus* subspecies (Additional file 1: Table S1) and representative sequences of closely related species as outgroups (e.g., *Mus spicilegus*—strain: ZBN). Haplotype network tree for MUS and MOL (*M. m. molossinus*) populations based on the mitochondrial *cytb* sequences in *Mus musculus* (**b**)

We combined the 23 *cytb* (1140 bp) gene sequences with a publicly accessible dataset of 169 *M. musculus* subspecies from Korea and other countries (Additional file 1: Table S1). The ML phylogeny from the *cytb* dataset was found to split into three discrete clades, corresponding to the CAS, DOM, and MUS subspecies, with BS/PP values > 95% (Fig. 3a). A well-differentiated cluster of MUS, including all Korean mice, was observed (Fig. 3a and b). Despite being previously designated as a distinct subspecies from hybrids between MUS and CAS in Japan, *M. m. molossinus* (MOL) was found to segregate within the MUS cluster. The topology of the median-joining network diagram that shows optimal connections between the haplotypes of the *M. musculus* subspecies also supported the existence of three discrete subspecies clusters (Fig. 4a). The Korean mice from the *cytb* haplotype network were shown to be confined within the MUS populations with unique haplotypes (e.g., *Hap*_1–3 and *Hap*_5–6) while also sharing some haplotypes in basal positions with Chinese populations (e.g., *Hap*_4 and *Hap*_7) (Fig. 4b; Additional file 1: Table S1). The shared haplotypes in the basal positions between Japanese and Korean (e.g., *Hap*_7–8) and Chinese (e.g., *Hap*_7) populations further support the close relationship with population expansion (Fig. 4b; Additional file 1: Table S1), representing a geographical distribution stretching from northern China over the Korean Peninsula into Japan. Additionally, MOL possesses four haplotypes (Fig. 4b; Additional file 1: Table S1) and shares a haplotype (e.g., *Hap*_7) with the MUS subspecies from China, Korea, and Japan, which is consistent with the observed clustering within the MUS sub-lineage (Fig. 3a).

The pairwise genetic distances (*p*-distance) between Korean mice and recognized subspecies populations ranged from 0.46% to 2.94%. Korean mice were the closest to the MUS lineage, including MOL from Japan, whereas the Korean mice were distantly related to CAS and DOM (Additional file 1: Table S3). The *Fst* values also supported the pairwise genetic differentiation among and within subspecific populations, indicating significant evolutionary differentiation between the three subspecies (Additional file 1: Table S4). The genetic variation between the MUS populations and Korean mice did not differ significantly (*Fst* = 0.188, *p* > 0.05). However, Korean mice within the MUS group remained distinct from the CAS (*Fst* = 0.902, *p* < 0.0001) and DOM populations (*Fst* = 0.889, *p* < 0.0001), although not significantly different from MOL (*Fst* = 0.490, *p* > 0.05) (Additional file 1: Table S4).

The genetic diversity and demographic statistics are summarized in Table 1. The G + C content in the *cytb* sequences was observed to be almost identical across *M. musculus* populations, ranging from 38.9% to 39.1%. Meanwhile, haplotype diversity (*Hd*) among MUS, CAS, and DOM ranged from 0.925 to 0.953, followed by MOL (0.711), with the smallest found in the Korean population (0.071). The nucleotide diversity (*π*) ranged from 0.44% in MUS to 0.51% in DOM, with the lowest in Korean mice (0.10%). Tajima’s *D* values, a statistical test of mutation neutrality, were not significant for most sample locations, except Southeast Asia. However, the negative values observed in the pooled samples from both MUS (-1.9628, *p* < 0.05) and CAS (-2.0617, *p* < 0.05) are indicative of a recent population expansion.

**Table 1:**
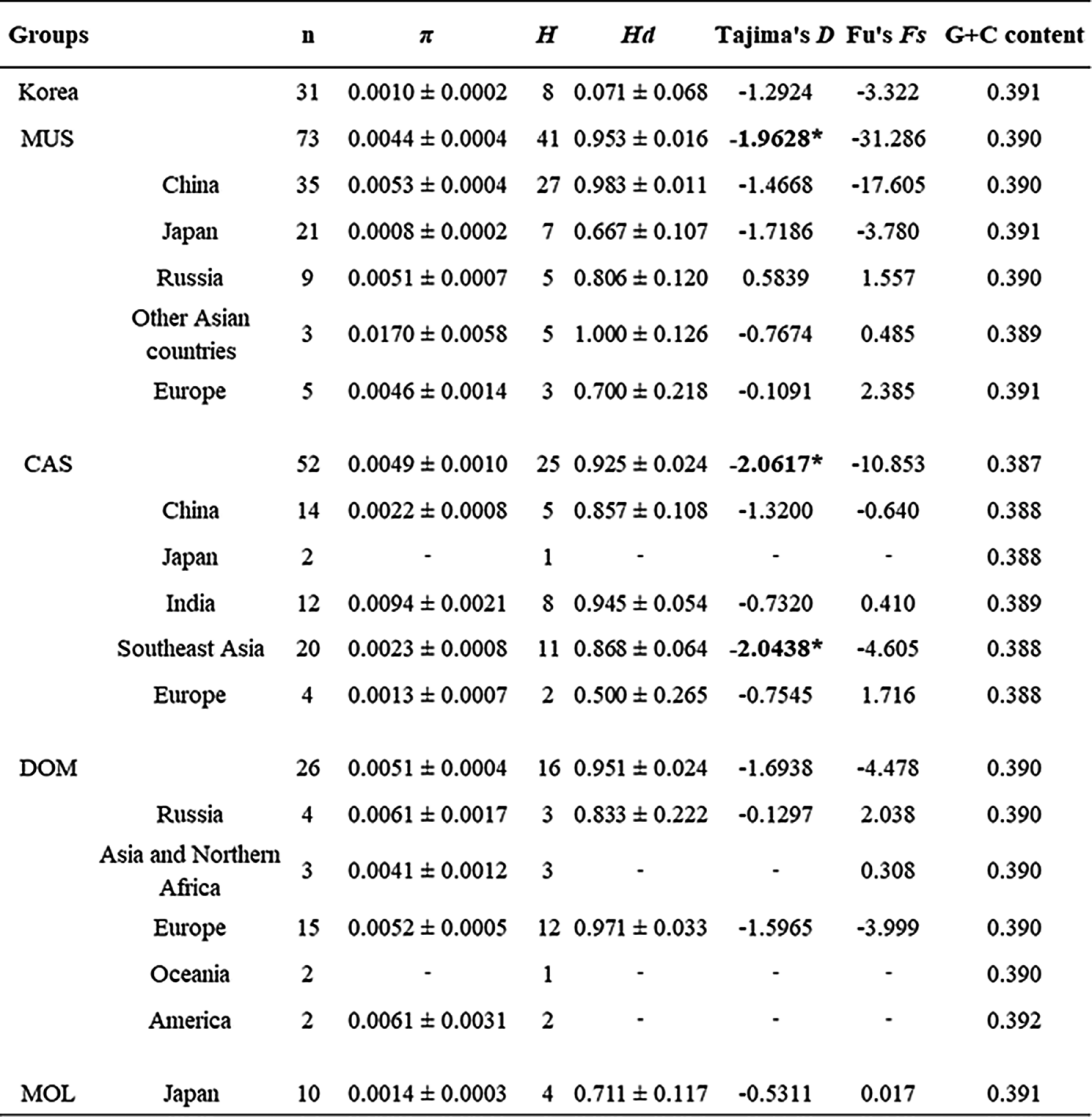
Genetic diversity and demographic statistics of *Mus musculus* subspecies based on mitochondrial *cytb* sequences

We used our 23 *cytb* (1140 bp) gene sequences with publicly accessible datasets of the 169 *M. musculus* subspecies (Additional file 1: Table S1). n, number of samples; *π*, nucleotide diversity; *H*, haplotype number; *Hd*, haplotype diversity; MUS, *M. m. musculus*; CAS, *M. m. castaneus*; DOM, *M. m. domesticus*; MOL, *M. m. molossinus*. Significant demographic parameters are indicated in bold font with an asterisk (*p < 0.05)

### Morphometrics

**Fig. 5:**
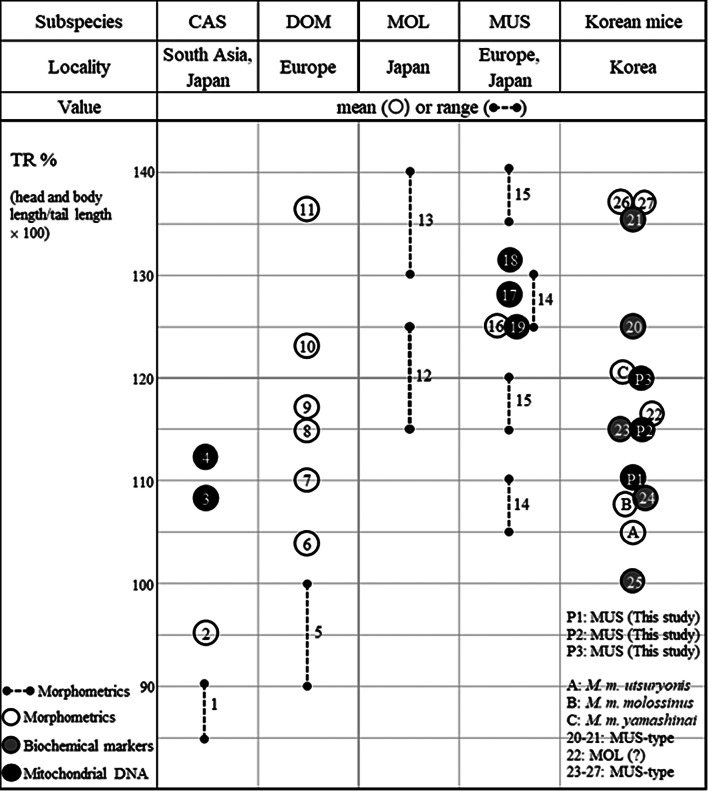
Comparison of tail ratio percentages (TR%) from the present Korean mice and formerly characterized subspecies populations. MUS, *M. m. musculus*; CAS, *M. m. castaneus*; DOM, *M. m. domesticus*; MOL, *M. m. molossinus*. Numbers in the figure indicate data from the references as shown in Additional file 1: Table S2: P1–P3 (this study), (A) [4], (B) [3], (C) [5], (1, 5, and 7–15) [2], (2, 6, and 16) [8], (3, 4, and 19) [17], (17 and 18) [18], (20 and 21) [14], (22) [9], (23–25) [10], (26 and 27) [11]

The haplotypes of the present Korean mice were shown to be MUS; meanwhile, the tail ratios from our samples and previously described subspecies populations are presented in Fig. 5 and Additional file 1: Table S2. The tail ratios for our Korean mice (ranging from 104.8 to 140.0%) were similar to those previously reported for Korean mice assigned to the subspecies of *M. m. molossinus* (107.7%) [2], *M. m. utsuryonis* (105.1%) [4], and *M. m. yamashinai* (121.7%) [5]. The CAS subspecies is characterized by a tail length that exceeds the head and body length [2]. This contradicts a recent molecular study showing tail ratios for CAS above 100% [17]. In contrast, the typical tail lengths for MUS and MOL are shorter than the head and body lengths [2], which aligns with our observations, including for Korean mice (Fig. 5).

**Fig. 6:**
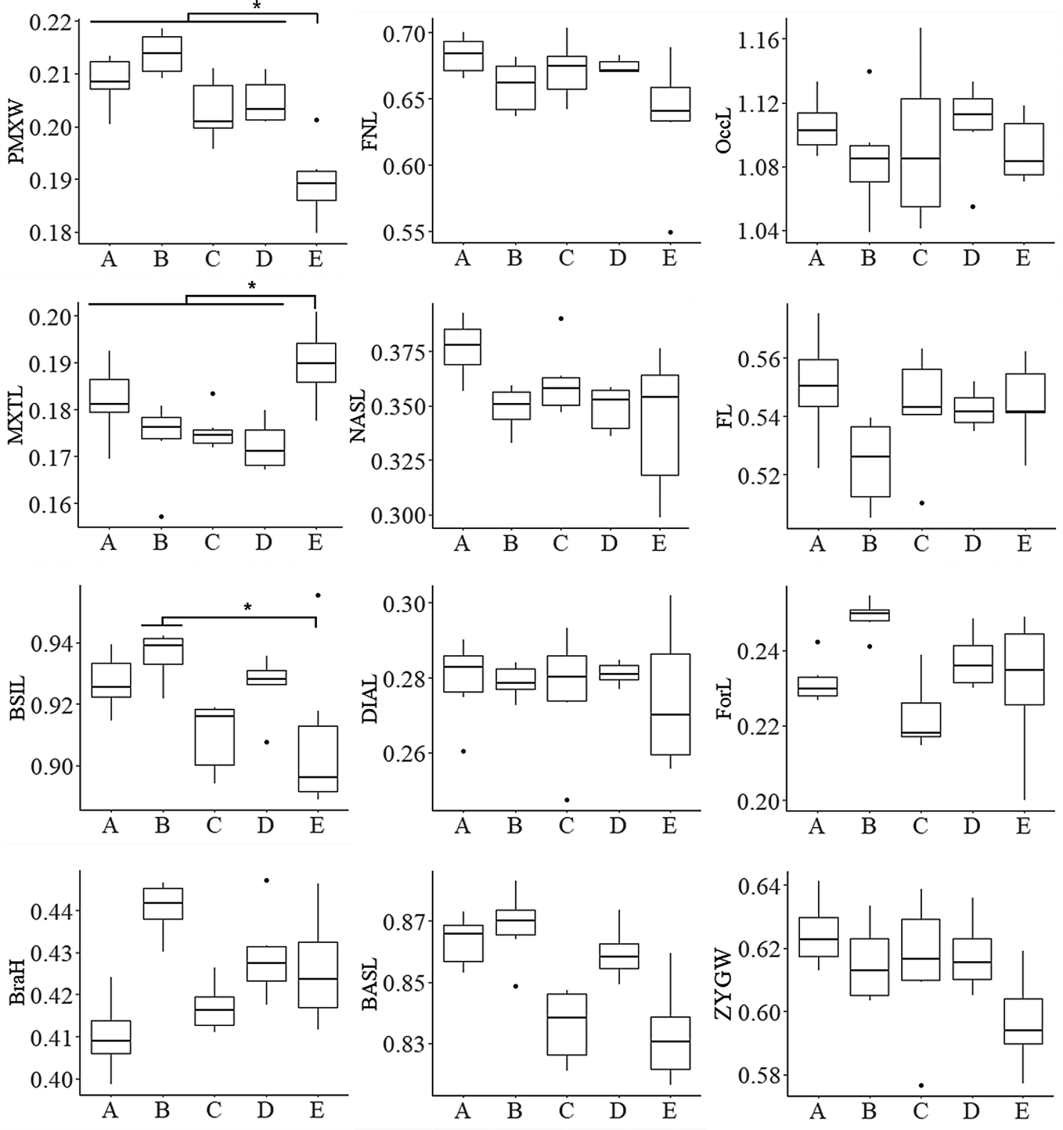
Box plot of craniometric variables with statistically significant levels among four commercially available laboratory mouse strains (CBA, C57BL/6H, C3H, and BALB/c derived from DOM) and our inbred mice from Goesan-gun (KG01BRA/1CNUf1–f3 and KG01BRA/1CNUm1–m3). No difference was observed for each variable (three males and three females, each at 9 weeks old) between genders in all the strains, including our inbred mice; thus, all mice with only adult specimens (six per strain; > 17.0 mm for the CBL of the skull [19]) were combined. The box plot shows the mean, range, and minimum and maximum values. A, CBA; B, C57BL/6H; C, C3H; D, BALB/c; E, our inbred mice from Goesan-gun. We used standardized craniometric indices to account for size differences between populations with only adult specimens (Y-axis). These indices are ratios relative to CBL and were log-transformed for the analysis

Using 3D micro-CT images, we measured linear distances between specific landmark points on the cranium (boundaries between different skull bones) to characterize the craniometric structure of Korean mice (Fig. 6). Standardized craniometric indices were used to account for size differences between populations with 9-week-old mice (only adult specimens with a skull CBL > 17.0 mm [19]). These indices are ratios relative to CBL and were log-transformed for analysis. The premaxillary tooth-patch width (PMXW) and maxillary teeth row length (MXTL) values indicate that Korean mice have narrower nasal bones and larger molars compared to the other strains derived from DOM (*p* < 0.05) (Fig. 6). Only basilar length (BSIL) among the other measured variables was statistically different among populations between the Korean mice and CH3 strains (*p* < 0.05) (Fig. 6). Nonetheless, despite not being statistically significant, the log-transformed craniofacial measurements of the frontonasal length (FNL), condylobasal length (CBL), nasal length (NASL), frontal bone length (FL), and basal length (BASL) in our Korean MUS mice appear smaller than those from several subspecies, including DOM, CAS (previously assigned *M. m. bactrianus*, now recognized as genetically indistinguishable from CAS [20], MUS, and other populations (e.g., *M. m. isatissus*) from the Middle East [21] (Additional file 1: Fig. S2). External measurements of nasal bone width (NW), NASL, and FNL likely appeared shorter in our Korean MUS and MOL (MSM/Ms) subspecies compared to other inbred mouse lines derived from DOM (BALB/c, C3H, C57BL/6H, CBA, ICR). However, the relative ratios of NW to NASL were comparable among these groups (Additional file 1: Fig. S3) [22]. These highly heritable craniometric features indicate that differences between subspecies are more pronounced than variation within subspecies, although genetically close strains do not always have morphologically identical crania.

**Fig. 7:**
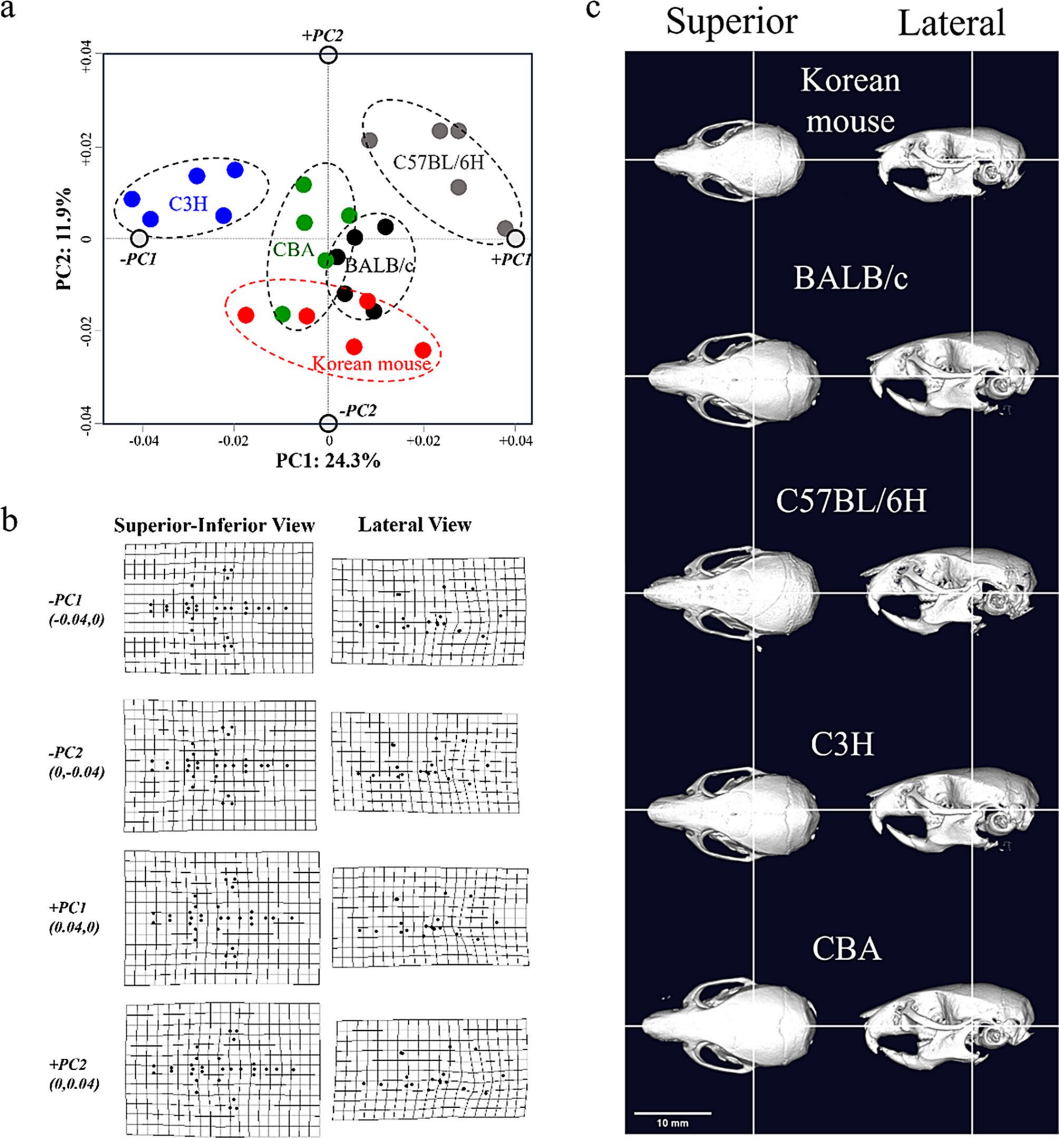
Analyses of cranial morphospaces among four laboratory mouse strains (CBA, C57BL/6H, C3H, and BALB/c derived from DOM) and our inbred mice from Goesan-gun (KG01BRA/1CNU). No differences were observed between genders in any variable (three males and two females, each at 9 weeks old) across all strains, including our inbred mice (five per strain). **a**) Principal component analysis (PCA) based on relative distances from the 30 digitized landmarks in the cranium; **b**) scatter plot of the relative coordinates after configuration of the 30 digitized landmarks in the cranium; **c**) micro-CT images in the superior view, with the calotte removed from the superior crania among four laboratory mouse strains and our inbred mouse from Goesan-gun

Morphospaces are visual representations of how craniometric variables define cranial shapes in space (Fig. 7). The 30 digitized landmarks on the cranium were compared to reveal craniofacial features between our specimens and four laboratory strains (Fig. 7). Therefore, landmark coordinates in the nasal region and surrounding frontal bones were further adjusted to ensure a good fit across all specimens and to account for the distinctive PMXW measurement. This reflects the Korean mouse having a relatively short and slender snout with a pointed tip. The maxillary contours also indicate that the tips of the alveolar processes (tooth sockets) are positioned slightly posteriorly compared to the more elaborate configuration in the DOM-derived laboratory strains. These represent subtle differences between the Korean mice and the other groups.

## Discussion

The key findings in this study are as follows: (1) Korean house mice represent populations of MUS with significantly lower genetic variation than other recognized subspecies. (2) The genetic structures, including *cytb* haplotype networks, suggest close phylogeographic relationships between Korea and neighboring countries, influencing MUS population dynamics. (3) There was no evidence supporting a unique subspecies designation for mice previously assigned to *M. m. molossinus*, *M. m. utsuryonis*, and *M. m. yamashinai*. These mice likely belong to existing MUS populations. Our findings support previous studies suggesting that the CAS subspecies found in Japan migrated directly from Southeast Asia to Japan without passing through Korea [7]. However, further studies with additional sampling and analyses in the southern regions of the Korean Peninsula and islands, including Jeju Island, are required to provide a robust validation of this hypothesis. As additional lineages may exist within Korea, we have also highlighted the need for future investigations into the potential presence of other genetic lineages in domestic populations.

The taxonomic status of Korean house mice has been a source of debate, with several subspecies assigned based on subtle morphological characteristics (e.g., MOL, *M. m. utsuryonis*, *M. m. yamashinai*, and MUS–like) or limited molecular markers (e.g., MOL-like, MUS-like, and MUS) (Fig. 5; Additional file 1: Table S2) [23]. Our analysis of publicly available genetic datasets, including samples from the type localities in the three nominal subspecies (*M. m. molossinus* [2, 3], *M. m. utsuryonis* [4], and *M. m. yamashinai* [5]), assigned all Korean house mice, including our samples, to the MUS populations. These populations exhibited remarkably low nucleotide diversity (*π* = 0.0010) and haplotype diversity (*hd* = 0.071) compared to mice from China (*π* = 0.0053; *hd* = 0.983), Russia (*π* = 0.0051; *hd* = 0.806), and Europe (*π* = 0.0046; *hd* = 0.700) (Table 1). The relatively low genetic variations among mtDNA sequences are likely due to a single lineage in the Korean Peninsula, perhaps established by limited gene flow within geographically restricted matrilines. However, whether these phylogeographic patterns are attributed to genetic drift following the migration of ancient populations or to regional differentiation within an isolated resident population warrants further consideration.

Among the 42 MUS haplotypes identified in China (27 haplotypes), Korea (8 haplotypes), and Japan (7 haplotypes), several basal positions were shared (e.g., *Hap*_4 and 7 between China and Korea; *Hap*_7 and 8 between Korea and Japan; *Hap*_7 among all three countries) (Fig. 4b; Additional file 1: Table S1). This supports the hypothesis that Korean MUS originated from a single lineage in northern China and has expanded into the Japanese Archipelago [6, 7, 24]. Tajima’s *D* and Fu’s *Fs* neutrality tests showed no significant deviations from neutrality for the MUS haplotypes in each location, except for Southeast Asia (Table 1), suggesting that these local populations are currently undergoing neutral evolution. However, pooling data across populations yielded a higher negative Tajima’s *D* value (-1.9628, *p* < 0.05), which could indicate a greater proportion of genetic variation within matrilines in MUS. Indeed, pooling data can signal rapid population expansion, range expansion, or purifying selection events leading to population subdivision. However, population structures in interactions with the sampling scheme and sample size can distort the interpretation of these neutrality tests and the associated reflection of regional demographic history.

While this study provides critical insights into the taxonomy of Korean house mice, certain limitations warrant consideration. The reliance on mtDNA (*cytb*) and a modest sample size constrain the resolution of population-level analyses. The absence of nuclear genomic data and comprehensive demographic modeling further limits the ability to disentangle evolutionary relationships. Notably, the low haplotype diversity (*hd* = 0.071) and Tajima’s *D* and Fu’s *Fs* values observed in Korean mice suggest potential influences of recent founder effects, population bottlenecks, or anthropogenic pressures, which remain unresolved. Our research team has recently completed whole-genome sequencing of the same Korean mouse samples, and the genomic results independently corroborate the *Cyt b* – based inferences (in preparation). Therefore, future research should prioritize three key directions: (1) expanding sampling efforts to encompass a wider geographic range, (2) integrating nuclear genomic data to complement mitochondrial findings, and (3) systematically investigating drivers of low genetic diversity, such as human-mediated dispersal, climate-associated adaptations, and historical demographic bottlenecks. Such efforts will clarify the interplay of genetic, ecological, and anthropogenic factors shaping the evolutionary trajectory of this commensal population.

Notably, Korean house mice have been classified under a standardized taxonomic framework for nearly a century, with researchers assigning three subspecies based on morphological traits (e.g., external body measurements including tail ratios, fur/hair color phenotypes): *M. m. molossinus* (Jeju Island), *M. m. utsuryonis* (Ulleung Island), and *M. m. yamashinai* (mainland Korean Peninsula) [2–5]. This system has been formally recognized in regional zoological literature and field guides [25], despite ongoing debates about the validity of applying the system for broader taxonomic contexts. The tail ratios (TRs) for our wild mice (range: 104.8–128.0%; mean: 115.4%) were comparable to previously reported values (range: 98.4–112.3%; mean: 105.1%) from Ulleung Island, suggesting that these mice belong to the same subspecific group and differ only following some natural variation. Similarly, the TRs of all our specimens (range: 104.8–140.0%) also overlapped with those reported for *M. m. molossinus* (107.7%), *M. m. utsuryonis* (105.1%), and *M. m. yamashinai* (121.7%) (Fig. 5; Additional file 1: Table S2). Based on our phylogeographic re-evaluation, Korean mice appear to represent a single lineage within MUS. This suggests that the previously assigned subspecies (e.g., *M. m. molossinus* on Jeju Island, *M. m. utsuryonis* on Ulleung Island, and *M. m. yamashinai* on the Korean Peninsula) from the associated type localities likely fall within the natural variation of MUS populations, even though the genetic data for these specific subspecies are unavailable. While the TR is a valuable tool for assessing general morphological features and mitigating age-related biases in mice [8, 18, 19], our data underscore that this trait alone cannot reliably distinguish subspecies, as intraspecific variation overlaps significantly across populations.

The TR values in this study were calculated using skeletal measurements: tail length (TL; anus to tail tip) and head-body length (HBL; snout to tail base) (Additional File 1: Fig. S1). This differs from traditional external TR protocols, which measure soft tissue. While pilot comparisons with preserved and fresh specimens revealed minor discrepancies (1–4%) between skeletal and external TR values, we prioritized methodological uniformity across samples to ensure internal validity. Although our formula (HBL/TL × 100) aligns with conventional TR calculations, applying this formula to skeletal elements rather than external morphology requires careful interpretation when comparisons are performed across studies. For example, external HBL measurements incorporate compressible soft tissue (e.g., skin, muscle), while skeletal HBL offers a fixed osteological reference. Thus, our TR analyses assessed the relative variation within the study samples rather than providing absolute benchmarks against prior datasets.

Cranial morphology in mice is shaped by genetic and epigenetic factors during development, rendering the cranial morphology a strongly heritable trait [22, 26, 27]. This study employed 3D micro-CT-derived landmark data to quantify craniofacial geometry through inter-landmark distance ratios, capturing the multidimensional complexity of this metric (Fig. 7). Our primary objective was to assess whether Korean house mice, historically classified *as M. m. molossinus*, *M. m. utsuryonis*, or *M. m. yamashinai*, exhibit genetic or morphological distinctiveness from the broader *M. m. musculus* lineage. Comparisons with *M. m. domesticus* (DOM)-derived inbred strains (CBA, C57BL/6H, C3H, BALB/c) served as a morphological benchmark, revealing pronounced craniofacial divergence: Korean mice display a shorter, narrower snout with a pointed rostral structure compared to DOM strains [27]. Meanwhile, we found no consistent evidence supporting the classification of Korean mice as a distinct subspecies when integrating the mitochondrial DNA analysis with craniometric data. Instead, the observed variations aligned with an adaptation within *M. m. musculus*. These results emphasize the necessity of combining genomic, morphological, and ecological datasets to resolve taxonomic uncertainties in phenotypically plastic taxa. Our findings advocate for revising legacy classifications to reflect evolutionary relationships rather than historical morphological assumptions.

## Conclusions

This study resolves the longstanding taxonomic debate surrounding Korean house mice, demonstrating that these mice constitute a single subspecific group within the *Mus musculus musculus* lineage rather than distinct subspecies. The observed cranial and morphological variations among Korean mice are explained by local adaptation rather than true taxonomic divergence. Therefore, by integrating comprehensive genetic and morphological analyses, our research resolves longstanding ambiguities regarding the taxonomic status of Korean house mice. These findings hold dual significance: clarifying the evolutionary trajectory of a key human commensal species in East Asia and establishing critical baselines for biomedical research utilizing wild-derived mouse models by ensuring accurate taxonomic identification and a clear understanding of genetic backgrounds. Ultimately, our results contribute to a deeper understanding of how ecological and historical factors shape the diversity and distribution of commensal mammals, with broad implications for evolutionary biology and taxonomy research.

## Supplementary Information

Below is the link to the electronic supplementary material.

Supplementary Material 1

## Data Availability

Data are available from the corresponding author upon reasonable request.
