# Supplementary material for: Molecular phylogeny and morphometric divergence of native Korean wild mice (Mus musculus)
Source: Lab Anim Res. 2026 Mar 25;42:8. doi: 10.1186/s42826-026-00269-7 (PMC13015121; doi:10.1186/s42826-026-00269-7)
Supplement: Supplementary file 1 — Supplementary Material 1 [file 42826_2026_269_MOESM1_ESM.docx]

**Supplementary Information (Additional file 1)**

**
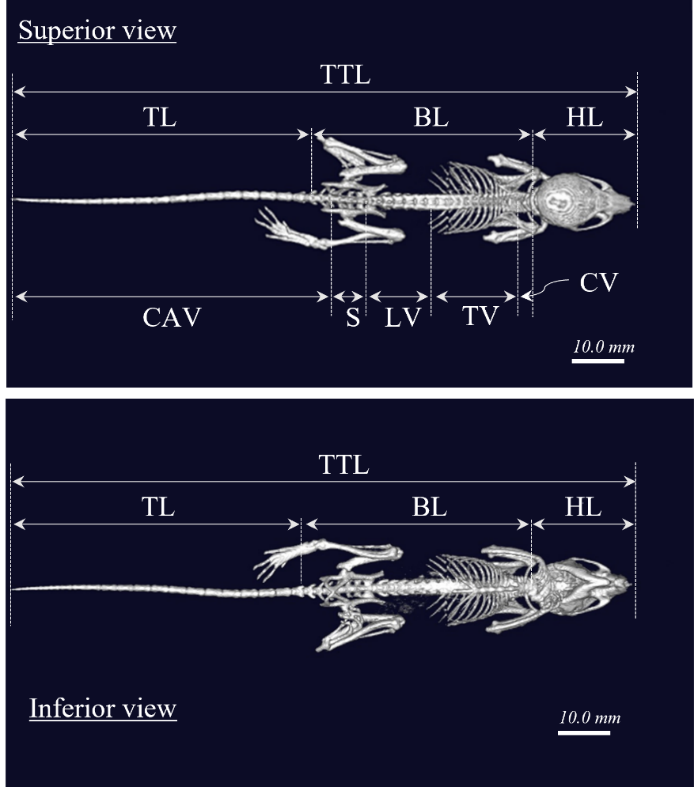
**

**Fig. S1** External measurements of the bone length on micro-CT images of a mouse. TTL, total tail length; TL, tail length from the anus to the tip of tail; HL, head length; BL, body length; HL + BL, head and body lengths determined from the snout to the anus; tail ratio: HL + BL / TL × 100); CV, cervical vertebrae; TV, thoracic vertebrae; LV, lumbar vertebrae; S, sacrum; CAV, caudal vertebrae.

**
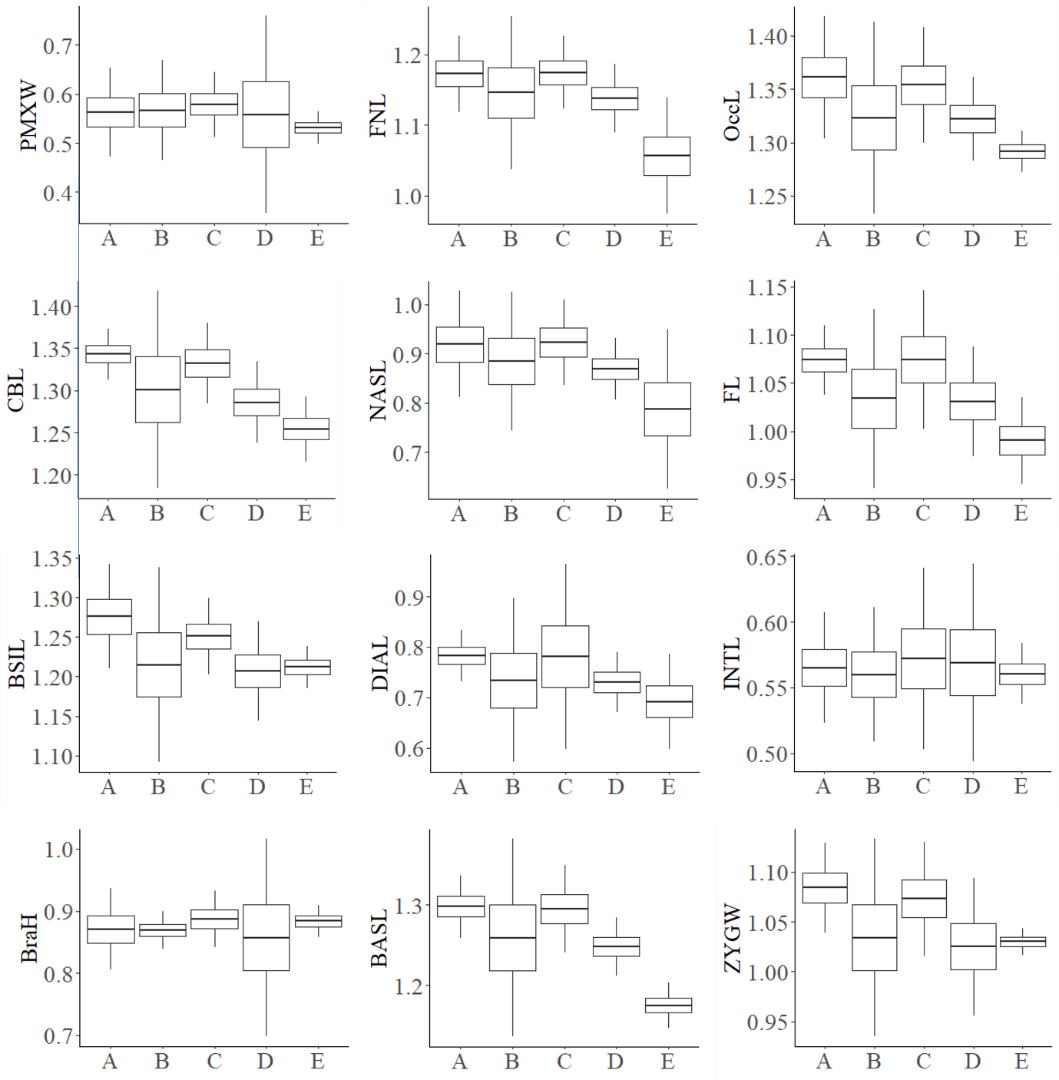
**

**Fig. S2** Comparison of craniometric variables between wild house mice from the Middle East [1] and our Korean inbred mice. All craniometric variables in the box plot are presented using a logarithmic scale. The box plot shows the mean, range, and minimum and maximum values. A, DOM; B, CAS (previously assigned to *M. m. bactrianus* but is genetically indistinguishable from CAS [2]); C, MUS; D, *M. m. isatissus*; E, our inbred mice from Goesan-gun.


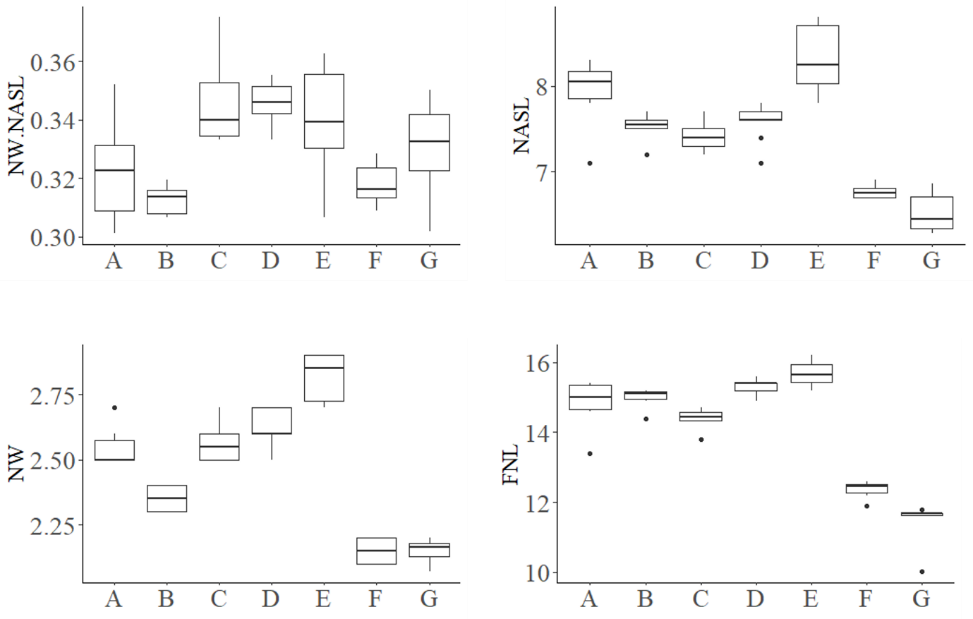


**Fig. S3** Comparison of craniometric variables among several inbred mice, including our Korean inbred mice. The box plot shows the mean, range, and minimum and maximum values. The data used for A–F are from [3]. A, C57BL/6J; B, BALB/Ca; C, C3H/HeJ; D, CBA/JNCrj; E, ICR; F, MSM/Ms; G, our inbred mice from Goesan-gun.

**Table S1** Information on samples: GenBank accession numbers for mitochondrial *cytb* sequences. ^+^ This study. MUS, *M. m. musculus*; CAS, *M. m. castaneus*; DOM, *M. m. domesticus*; MOL, *M. m. molossinus*.

|  | **Subspecies** | **Locality** |  | **Sample Name** | **Gene Bank  accession No.** | **Haplotype No.** |
| --- | --- | --- | --- | --- | --- | --- |
| 1 | MUS | Korea^+^ | Goesan | GS01 | OQ506519.1 | Hap_1 |
| 2 | MUS |  |  | GS02 | OQ506520.1 | Hap_1 |
| 3 | MUS |  |  | GS03 | OQ506521.1 | Hap_1 |
| 4 | MUS |  |  | GS04 | OQ506522.1 | Hap_1 |
| 5 | MUS |  |  | GS05 | OQ506523.1 | Hap_1 |
| 6 | MUS |  |  | GS06 | OQ506524.1 | Hap_1 |
| 7 | MUS |  |  | GS07 | OQ506525.1 | Hap_1 |
| 8 | MUS |  |  | GS08 | OQ506526.1 | Hap_1 |
| 9 | MUS |  |  | GS09 | OQ506527.1 | Hap_1 |
| 10 | MUS |  |  | GS10 | OQ506528.1 | Hap_1 |
| 11 | MUS |  |  | GS11 | OQ506529.1 | Hap_1 |
| 12 | MUS |  |  | GS12 | OQ506530.1 | Hap_2 |
| 13 | MUS |  | Ulleung | UL01 | OQ506531.1 | Hap_1 |
| 14 | MUS |  |  | UL02 | OQ506532.1 | Hap_1 |
| 15 | MUS |  |  | UL03 | OQ506533.1 | Hap_1 |
| 16 | MUS |  |  | UL04 | OQ506534.1 | Hap_1 |
| 17 | MUS |  | Naju | NJ01 | OQ506535.1 | Hap_3 |
| 18 | MUS |  | Goheung | GH01 | OQ506536.1 | Hap_4 |
| 19 | MUS |  | Wanju | WJ01 | OQ506537.1 | Hap_4 |
| 20 | MUS |  | Yeongju | YJ01 | OQ506538.1 | Hap_5 |
| 21 | MUS |  |  | YJ02 | OQ506539.1 | Hap_5 |
| 22 | MUS |  | Gageo Island | GG01 | OQ506540.1 | Hap_4 |
| 23 | MUS |  | Jeju island | JJ01 | AO53266.1 | Hap_6 |
| 24 | MUS |  | Ganghwa | HS4238 | AB649562.1 | Hap_4 |
| 25 | MUS |  | Hwacheon | HS4235 | AB649561.1 | Hap_7 |
| 26 | MUS |  |  | HS4233 | AB649559.1 | Hap_4 |
| 27 | MUS |  |  | HS4234 | AB649560.1 | Hap_4 |
| 28 | MUS |  | Peklyong Island | HS682 | AB205275.1 | Hap_7 |
| 29 | MUS |  | Busan | MG444 | AB649558.1 | Hap_8 |
| 30 | MUS |  | Suwon | MG5018 | AB649556.1 | Hap_4 |
| 31 | MUS |  |  | MG5017 | AB649555.1 | Hap_4 |
| 32 | MUS | Afghanistan | Kabul | BRC3566 | AB649510.1 | Hap_45 |
| 33 | MUS | China | Akus | MG608 | AB649524.1 | Hap_33 |
| 34 | MUS |  | Baodi | MG805 | AB649543.1 | Hap_16 |
| 35 | MUS |  |  | MG928 | AB649542.1 | Hap_11 |
| 36 | MUS |  | Beijing | MG5066 | AB649525.1 | Hap_32 |
| 37 | MUS |  | Dunhuang | MG871 | AB649549.1 | Hap_13 |
| 38 | MUS |  | Golmud | MG870 | AB649548.1 | Hap_14 |
| 39 | MUS |  |  | MG869 | AB649547.1 | Hap_14 |
| 40 | MUS |  | Hohhot | MG851 | AB649546.1 | Hap_13 |
| 41 | MUS |  |  | MG808 | AB649545.1 | Hap_15 |
| 42 | MUS |  | Hulin | MG835 | AB820921.1 | Hap_10 |
| 43 | MUS |  |  | MG919 | AB649552.1 | Hap_10 |
| 44 | MUS |  |  | MG918 | AB649551.1 | Hap_11 |
| 45 | MUS |  |  | MG920 | AB649553.1 | Hap_27 |
| 46 | MUS |  | Jiayuguan | MG5086 | AB649522.1 | Hap_32 |
| 47 | MUS |  | Jixian | MG806 | AB649544.1 | Hap_26 |
| 48 | MUS |  | Laiyang | MG969 | AB649535.1 | Hap_19 |
| 49 | MUS |  |  | MG967 | AB649533.1 | Hap_19 |
| 50 | MUS |  |  | MG966 | AB649532.1 | Hap_20 |
| 51 | MUS |  |  | MG964 | AB649531.1 | Hap_19 |
| 52 | MUS |  |  | MG970 | AB649536.1 | Hap_29 |
| 53 | MUS |  |  | MG968 | AB649534.1 | Hap_30 |
| 54 | MUS |  |  | MG963 | AB649530.1 | Hap_31 |
| 55 | MUS |  | Lhasa | MG723 | AB819907.1 | Hap_9 |
| 56 | MUS |  | Manasi | MG597 | AB649523.1 | Hap_25 |
| 57 | MUS |  | Qiqiha | MG991 | AB649539.1 | Hap_18 |
| 58 | MUS |  |  | MG992 | AB649540.1 | Hap_7 |
| 59 | MUS |  | Shijiazhuang | MG978 | AB649538.1 | Hap_11 |
| 60 | MUS |  | Tongliao | MG812 | AB649550.1 | Hap_12 |
| 61 | MUS |  | Tumen | MG980 | AB649557.1 | Hap_4 |
| 62 | MUS |  |  | MG875 | AB649528.1 | Hap_22 |
| 63 | MUS |  |  | MG874 | AB649527.1 | Hap_23 |
| 64 | MUS |  |  | MG867 | AB649526.1 | Hap_24 |
| 65 | MUS |  | Xiaogan | MG956 | AB649529.1 | Hap_21 |
| 66 | MUS |  | Zhengzhou | MG927 | AB649541.1 | Hap_17 |
| 67 | MUS |  |  | MG971 | AB649537.1 | Hap_28 |
| 68 | MUS | Czech Republic | Lhotka | HS3948 | AB819918.1 | Hap_48 |
| 69 | MUS | Estonia | Tallinn | MG3044 | AB649507.1 | Hap_47 |
| 70 | MUS | Japan | Fukuoka | HS4272 | AB649582.1 | Hap_34 |
| 71 | MUS |  | Hokkaido | HS4411 | AB649564.1 | Hap_7 |
| 72 | MUS |  |  | HS4271 | AB649563.1 | Hap_8 |
| 73 | MUS |  | Ibaraki | HS4169 | AB649566.1 | Hap_7 |
| 74 | MUS |  | Kagoshima | BRC3515 | AB649573.1 | Hap_38 |
| 75 | MUS |  |  | MAI-489 | AB649571.1 | Hap_7 |
| 76 | MUS |  |  | MAI-491 | AB649570.1 | Hap_8 |
| 77 | MUS |  |  | MAI-487 | AB649569.1 | Hap_7 |
| 78 | MUS |  |  | MAI-488 | AB649568.1 | Hap_7 |
| 79 | MUS |  |  | MAI-492 | AB649567.1 | Hap_7 |
| 80 | MUS |  | Kyoto | MG422 | AB649572.1 | Hap_7 |
| 81 | MUS |  | Sado | HS4246 | AB649565.1 | Hap_36 |
| 82 | MUS |  | Tokushima | HS4121 | AB649581.1 | Hap_7 |
| 83 | MUS |  |  | HS4120 | AB649580.1 | Hap_7 |
| 84 | MUS |  | Tottori | HS4099 | AB649579.1 | Hap_7 |
| 85 | MUS |  |  | HS4098 | AB649578.1 | Hap_7 |
| 86 | MUS |  |  | HS4097 | AB649577.1 | Hap_7 |
| 87 | MUS |  | Yamaguchi | HS4275 | AB649576.1 | Hap_35 |
| 88 | MUS |  |  | HS4274 | AB649575.1 | Hap_35 |
| 89 | MUS |  |  | HS4273 | AB649574.1 | Hap_35 |
| 90 | MUS |  |  | HS4412 | AB649583.1 | Hap_37 |
| 91 | MUS | Kazakhstan | Aktobe | HS1464 | AB649511.1 | Hap_25 |
| 92 | MUS | Moldova | Ungeni | HS4326 | AB649516.1 | Hap_41 |
| 93 | MUS |  |  | HS4325 | AB649515.1 | Hap_41 |
| 94 | MUS | Russia | GornoAltaysk | HS3605 | AB649519.1 | Hap_40 |
| 95 | MUS |  | Irkutsk | HS3608 | AB649518.1 | Hap_40 |
| 96 | MUS |  | Komsomolsk-on-Amure | MG3042 | AB819919.1 | Hap_40 |
| 97 | MUS |  |  | MG3041 | AB819915.1 | Hap_40 |
| 98 | MUS |  | Novi | MG3012 | AB819916.1 | Hap_39 |
| 99 | MUS |  | Primorye | HS1337 | AB649521.1 | Hap_41 |
| 100 | MUS |  |  | HS1413 | AB649520.1 | Hap_42 |
| 101 | MUS |  |  | HS3612 | AB649509.1 | Hap_39 |
| 102 | MUS |  | Sulak | HS4327 | AB649517.1 | Hap_43 |
| 103 | MUS | Ukraine | Donetsk | MG3065 | AB819917.1 | Hap_41 |
| 104 | MUS | Uzbekistan | Tashkent | HS1338 | AB649512.1 | Hap_46 |
| 105 | CAS | Bangladesh | Comilla District | HS3701 | AB820905.1 | Hap_53 |
| 106 | CAS |  |  | HS3689 | AB820904.1 | Hap_61 |
| 107 | CAS |  |  | HS2925 | AB820903.1 | Hap_62 |
| 108 | CAS |  |  | HS2924 | AB649497.1 | Hap_69 |
| 109 | CAS | China | Kunming | HS507 | AB649502.1 | Hap_49 |
| 110 | CAS |  |  | HS506 | AB649501.1 | Hap_49 |
| 111 | CAS |  | Manzhouli | MG863 | AB649505.1 | Hap_55 |
| 112 | CAS |  | Chongqing | MG654 | AB820919.1 | Hap_56 |
| 113 | CAS |  | Guangzhou | MG504 | AB820918.1 | Hap_56 |
| 114 | CAS |  |  | MG503 | AB820917.1 | Hap_56 |
| 115 | CAS |  | Guilin | MG501 | AB820916.1 | Hap_57 |
| 116 | CAS |  | Hainan Island | MG712 | AB649504.1 | Hap_68 |
| 117 | CAS |  | Hulin | MG834 | AB820920.1 | Hap_55 |
| 118 | CAS |  | Kunming | HS512 | AB819914.1 | Hap_49 |
| 119 | CAS |  |  | HS509 | AB819913.1 | Hap_49 |
| 120 | CAS |  |  | HS508 | AB649503.1 | Hap_49 |
| 121 | CAS |  | Ningpo | MG786 | AB819920.1 | Hap_50 |
| 122 | CAS |  | Shanghai | MG438 | AB820913.1 | Hap_58 |
| 123 | CAS | Georgia | Sakhalin | MG3047 | AB820911.1 | Hap_44 |
| 124 | CAS |  |  | MG3046 | AB820910.1 | Hap_59 |
| 125 | CAS |  | Vladivostok | MG3077 | AB820912.1 | Hap_59 |
| 126 | CAS |  |  | MG3023 | AB820909.1 | Hap_59 |
| 127 | CAS | India | Bhubaneswar | HI302 | AB649491.1 | Hap_71 |
| 128 | CAS |  | Coimbatore | BRC3026 | AB820897.1 | Hap_52 |
| 129 | CAS |  | Delhi | HI187 | AB649490.1 | Hap_72 |
| 130 | CAS |  |  | HI186 | AB820901.1 | Hap_56 |
| 131 | CAS |  |  | BRC3015 | AB819908.1 | Hap_44 |
| 132 | CAS |  | Ghaziabad | BRC3025 | AB819912.1 | Hap_51 |
| 133 | CAS |  | Kolkata | BRC3017 | AB819910.1 | Hap_53 |
| 134 | CAS |  | Leh | HI159 | AB649489.1 | Hap_66 |
| 135 | CAS |  |  | HI161 | AB649488.1 | Hap_66 |
| 136 | CAS |  | Maharashtra | BRC3016 | AB819909.1 | Hap_54 |
| 137 | CAS |  | Mysore | HS4173 | AB649487.1 | Hap_56 |
| 138 | CAS |  | Pachmarhi | BRC3018 | AB819911.1 | Hap_52 |
| 139 | CAS | Indonesia | Bali | HI116 | AB820899.1 | Hap_56 |
| 140 | CAS |  | Flores | HS3736 | AB820908.1 | Hap_56 |
| 141 | CAS |  | Java | HI134 | AB820900.1 | Hap_63 |
| 142 | CAS |  |  | HI111 | AB820898.1 | Hap_56 |
| 143 | CAS |  | Maluku Province | HS4112 | AB649499.1 | Hap_56 |
| 144 | CAS | Japan | Fukushima | MG489 | AB820915.1 | Hap_49 |
| 145 | CAS |  |  | MG488 | AB820914.1 | Hap_49 |
| 146 | CAS | Myanmar | Lashio | HS3357 | AB649498.1 | Hap_56 |
| 147 | CAS |  |  | HS3721 | AB820907.1 | Hap_56 |
| 148 | CAS |  | Mt Popa | HS3720 | AB820906.1 | Hap_60 |
| 149 | CAS | Pakistan | Islamabad | HI175 | AB649486.1 | Hap_67 |
| 150 | CAS | Philippines | Catarman | HI196 | AB820902.1 | Hap_62 |
| 151 | CAS | Philippines | Quezon City | BRC3528 | AB649500.1 | Hap_64 |
| 152 | CAS | Sri Lanka | Colombo | HI485 | AB649495.1 | Hap_65 |
| 153 | CAS |  |  | HI488 | AB649496.1 | Hap_56 |
| 154 | CAS |  | Peradeniya | HI483 | AB649493.1 | Hap_65 |
| 155 | CAS |  |  | HI481 | AB649492.1 | Hap_65 |
| 156 | CAS |  |  | HI484 | AB649494.1 | Hap_70 |
| 157 | DOM | Australia | New South Wales | ANWC-M29996 | AB649475.1 | Hap_79 |
| 158 | DOM |  |  | ANWC-M29995 | AB649474.1 | Hap_79 |
| 159 | DOM | Canada | Rocheleau | MG414 | AB649477.1 | Hap_79 |
| 160 | DOM | Cyprus | Agios Theodoros | IZEA1666 | AB649471.1 | Hap_81 |
| 161 | DOM |  | Maroni | IZEA1662 | AB649469.1 | Hap_81 |
| 162 | DOM |  | Nicosia | IZEA1642 | AB649470.1 | Hap_82 |
| 163 | DOM | France | Montpellier | MG411 | AB649459.1 | Hap_80 |
| 164 | DOM | Germany | Kubelhof | HS3959 | AB649455.1 | Hap_88 |
| 165 | DOM |  | Weidesgrun | HS3958 | AB649456.1 | Hap_79 |
| 166 | DOM | Greece | Crete | IZEA2039 | AB649467.1 | Hap_84 |
| 167 | DOM |  |  | IZEA2027 | AB649466.1 | Hap_84 |
| 168 | DOM |  |  | IZEA2026 | AB649465.1 | Hap_85 |
| 169 | DOM |  | Epanomi | IZEA3919 | AB649468.1 | Hap_83 |
| 170 | DOM | Iran | Ahvaz | NIG934 | AB649479.1 | Hap_77 |
| 171 | DOM | Italy | Milan | MG395 | AB649462.1 | Hap_86 |
| 172 | DOM |  | Aosta | HS589 | AB649460.1 | Hap_87 |
| 173 | DOM |  | Himera Sicily | IZEA2946 | AB649461.1 | Hap_77 |
| 174 | DOM | Peru | Coppeck | BRC3647 | AB649478.1 | Hap_78 |
| 175 | DOM | Philippines | Manila | HS3882 | AB649484.1 | Hap_73 |
| 176 | DOM | Russia | Amur River mouth | HS1466 | AB649481.1 | Hap_75 |
| 177 | DOM |  | Moscow | MG3055 | AB649480.1 | Hap_76 |
| 178 | DOM |  | Sakhalin | HS3607 | AB649482.1 | Hap_75 |
| 179 | DOM |  | Tomsk | HS3604 | AB649483.1 | Hap_74 |
| 180 | DOM | Tunisia | Monastir | MG393 | AB649473.1 | Hap_80 |
| 181 | DOM | Turkey | Altindere | IZEA6089 | AB649464.1 | Hap_75 |
| 182 | DOM |  |  | IZEA6068 | AB649463.1 | Hap_75 |
| 183 | MOL | Japan | Asahikawa | KT3285 | AB205283.1 | Hap_93 |
| 184 | MOL |  | Chiba | HS2470 | AB205314.1 | Hap_90 |
| 185 | MOL |  |  | HS2469 | AB205313.1 | Hap_90 |
| 186 | MOL |  |  | HS2471 | AB205315.1 | Hap_90 |
| 187 | MOL |  | Fukagawa | HS2324 | AB205284.1 | Hap_93 |
| 188 | MOL |  |  | HS2325 | AB205285.1 | Hap_93 |
| 189 | MOL |  | Kanazawa | HS2814 | AB205316.1 | Hap_7 |
| 190 | MOL |  |  | HS2815 | AB205317.1 | Hap_91 |
| 191 | MOL |  |  | HS2816 | AB205318.1 | Hap_7 |
| 192 | MOL |  |  | HS2817 | AB205319.1 | Hap_7 |
| 193 | MOL |  | Miyazaki | KT3226 | AB205321.1 | Hap_7 |
| 194 | MOL |  |  | KT3225 | AB205320.1 | Hap_7 |
| 195 | MOL |  | Nayoro | HS2326 | AB205281.1 | Hap_93 |
| 196 | MOL |  |  | HS2327 | AB205282.1 | Hap_93 |
| 197 | MOL |  | Okinawa | HS2788 | AB205322.1 | Hap_92 |
| 198 | MOL |  | Otaru | HS2340 | AB205291.1 | Hap_89 |
| 199 | MOL |  | Takikawa | HS2444 | AB205286.1 | Hap_93 |

**Table S2** Tail ratios (head and body length / tail length × 100) of wild house mice from the present study and formerly characterized subspecies populations.

|  | **Method** | **Locality** | **Type locality** | **Tail ratio (%)** | | **Remarks** | **Reference** |
| --- | --- | --- | --- | --- | --- | --- | --- |
|  |  |  |  | **Mean** | **Range** |  |  |
| **MUS** | a | Sweden, Estonia | Upsala, Sweden |  | 115–120 | Variation: males, 92–143; females, 97–155 | [4] |
| **(Linnaeus, 1758)** |  |  |  |  | 135–140 |  |  |
|  | a | East Prussia, Hungary |  |  | 105–110 |  |  |
|  |  | **Korea** |  |  |  |  |  |
| *M. m. yamashinai* | a | Mokpo and its neighboring islands, and inlands | Mokpo, Korea | 121.7 (n =10) | 103.5–136.2 | Originally *M. bactrianus yamashinai* | [5] |
| *M. m. molossinus* | a | Jeju-do (islands) | Jeju, Korea | 107.7 |  |  | [4,6] |
| *M. m. utsuryonis* | a | Ulleung-do (islands) | Ulleung, Korea | 105.1 (n = 5) | 98.4–112.3 | Originally *M. molossinus utsuryonis* | [7] |
| MUS | b | Geosan |  | 111.1 (n = 6) | 109.0–113.7 | Rice field and granaries | This study |
| MUS | b | Yeongju, Wanju |  | 120.8 (n = 3) | 105.1–140.0 | Mountain areas |  |
| MUS | b | Ulleung-do, Gageo-do |  | 115.4 (n = 4) | 104.8–128.0 | Islands |  |
| MUS-like | c | Chuncheon |  | 125.6 (males) |  | Based on AKV-type murine leukemia virus gene | [8] |
|  | c |  |  | 136 (females) |  |  |  |
| - | a | Chuncheon |  | 116.0 |  | Consistent with *M. m. yamashinai* | [9] |
| MUS-like | c | Chuncheon |  | 115.1 |  |  | [10] |
| MUS-like | c | Sokcho |  | 108.4 |  |  |  |
| MUS-like | c | Heongsung |  | 100.1 |  |  |  |
| MUS-like | a | Anmyun-do (islands) |  | 137.1 |  |  | [11] |
| MUS-like | a | Gumi |  | 137.6 |  |  |  |
| MUS | b | Honshu, Japan |  | 125.0 |  | *cytb* | [12] |
| MUS | b | Miura, Japan |  | 132.2 |  | *cytb* | [13] |
| MUS | b | Fujisawa, Japan |  | 127.5 |  | *cytb* |  |
| MUS | a | Northern Europe |  | 125.3 |  |  | [14] |
| **CAS** | a | South Asia | Philippine Islands |  | 85–90 |  | [4] |
| **(Waterhouse, 1843)** | b | Hokkaido, Japan |  | 108.4 |  | *cytb* | [12] |
|  | b | Honshu, Japan |  | 112.4 |  | *cytb* |  |
|  | a | Southeastern Asia |  | 95.6 |  |  | [14] |
| **DOM** | a | Switzerland | Dublin, Ireland | 115 |  |  | [4] |
| **(Rutty, 1772)** | a | France |  | 110 |  |  |  |
|  | a | Ireland |  | 123 |  |  |  |
|  | a | Shetland Islands |  | 117 |  |  |  |
|  | a | St. Kilda |  | 136 |  |  |  |
|  | a | Unknown |  |  | 90–100 |  |  |
|  | a | Europe |  | 104.0 |  |  | [14] |
| **MOL** | a | Japan | Nagasaki/Kyushu, Japan |  | 115–125 (males) | Variation: males, 119–138; females, 120–162 | [4] |
| **(Temminck, 1845)** | a |  |  |  | 130–140 (females) |  |  |

a (morphometrics), b (mitochondrial DNA), c (biochemical markers). MUS, *M. m. musculus*; CAS, *M. m. castaneus*; DOM, *M. m. domesticus*; MOL, *M. m. molossinus*.

**Table S3** Genetic distances (*p*-distance ± SD; %) within and between the *Mus musculus* subspecies based on mitochondrial *cytb* sequences.

| **Subspecies** | | **Sites** | **n** | **MUS** | | | | | | | **CAS** | **DOM** | **MOL** |
| --- | --- | --- | --- | --- | --- | --- | --- | --- | --- | --- | --- | --- | --- |
|  |  |  |  | **a** | **b** | **c** | **d** | **e** | **f** | **(b + c + d + e + f)** |  |  |  |
| **MUS** | **a** | Korea | 31 | 0.10 ± 0.08 |  |  |  |  |  |  |  |  |  |
|  | **b** | China | 35 | 0.51 ± 0.21 | 0.57 ± 0.30 |  |  |  |  |  |  |  |  |
|  | **c** | Japan | 21 | 0.18 ± 0.08 | 0.47 ± 0.26 | 0.08 ± 0.07 |  |  |  |  |  |  |  |
|  | **d** | Russia | 9 | 0.53 ± 0.16 | 0.58 ± 0.27 | 0.50 ± 0.24 | 0.51 ± 0.35 |  |  |  |  |  |  |
|  | **e** | Other Asian countries | 3 | 1.11 ± 0.98 | 1.12 ± 0.94 | 1.07 ± 0.96 | 1.10 ± 1.01 | 1.70 ± 1.21 |  |  |  |  |  |
|  | **f** | Europe | 5 | 0.56 ± 0.06 | 0.67 ± 0.26 | 0.44 ± 0.06 | 0.62 ± 0.32 | 1.27 ± 1.21 | 0.46 ± 0.32 |  |  |  |  |
|  | **(b + c + d + e + f)** | Non-Korean | 73 | 0.46 ± 0.38 | - | - | - | - | - | 0.58 ± 0.49 |  |  |  |
| **CAS** |  | - | 52 | 2.94 ± 0.10 | 2.82 ± 0.16 | 2.84 ± 0.11 | 2.90 ± 0.14 | 2.45 ± 1.06 | 2.97 ± 0.16 | 2.83 ± 0.33 | 0.41 ± 0.48 |  |  |
| **DOM** |  | - | 26 | 2.50 ± 0.16 | 2.51 ± 0.21 | 2.40 ± 0.15 | 2.57 ± 0.22 | 2.84 ± 0.32 | 2.47 ± 0.15 | 2.50 ± 0.23 | 3.28 ± 0.15 | 0.50 ± 0.20 |  |
| **MOL** |  | Japan | 10 | 0.22 ± 0.10 | 0.51 ± 0.27 | 0.12 ± 0.11 | 0.54 ± 0.25 | 0.65 ± 0.24 | 0.51 ± 0.09 | 0.45 ± 0.42 | 2.96 ± 0.13 | 2.51 ± 0.17 | 0.13 ± 0.11 |

We used our 23 *cytb* (1140 bp) sequences with the publicly accessible dataset of 169 *M. musculus* subspecies (Table 4). MUS, *M. m. musculus*; CAS, *M. m. castaneus*; DOM, *M. m. domesticus*; MOL, *M. m. molossinus*.

**Table S4** The pairwise *Fst* values for genetic differentiation among and within subspecific populations based on mitochondrial *cytb* sequences of *Mus musculus*.

| **Subspecies** | | **Sites** | **n** | **MUS** | | | | | | | **CAS** | **DOM** | **MOL** |
| --- | --- | --- | --- | --- | --- | --- | --- | --- | --- | --- | --- | --- | --- |
|  |  |  |  | **a** | **b** | **c** | **d** | **e** | **f** | **(b + c + d + e + f)** |  |  |  |
| **MUS** | **a** | Korea | 31 |  |  |  |  |  |  |  |  |  |  |
|  | **b** | China | 35 | 0.370 |  |  |  |  |  |  |  |  |  |
|  | **c** | Japan | 21 | 0.583 | 0.264 |  |  |  |  |  |  |  |  |
|  | **d** | Russia | 9 | 0.603 | 0.069 | 0.517 |  |  |  |  |  |  |  |
|  | **e** | Other Asian countries | 3 | 0.495 | 0.111 | 0.470 | 0.051 |  |  |  |  |  |  |
|  | **f** | Europe | 5 | 0.721 | 0.659 | 0.699 | 0.277 | 0.161 |  |  |  |  |  |
|  | **(b + c + d + e + f)** | Non-Korean | 73 | 0.188 | - | - | - | - | - |  |  |  |  |
| **CAS** |  | - | 52 | 0.902 | 0.827 | 0.891 | 0.848 | 0.739 | 0.860 | 0.902 |  |  |  |
| **DOM** |  | - | 26 | 0.889 | 0.784 | 0.869 | 0.803 | 0.732 | 0.803 | 0.889 | 0.864 |  |  |
| **MOL** |  | Japan | 10 | 0.490 | 0.297 | 0.318 | 0.273 | 0.231 | 0.288 | 0.490 | 0.558 | 0.659 |  |

We used 23 *cytb* (1140 bp) sequences with the publicly accessible dataset of 169 *M. musculus* subspecies (Table 4). MUS, *M. m. musculus*; CAS, *M. m. castaneus*; DOM, *M. m. domesticus*; MOL, *M. m. molossinus*.

**References**

1. Hamid HS, Darvish J, Rastegar-Pouyani ER, Mahmoudi A. Subspecies differentiation of the house mouse *Mus musculus* Linnaeus, 1758 in the center and east of the Iranian plateau and Afghanistan. Mammalia. 2016;1-22.
2. Lawal RA, Mathis VL, Barter ME, Charette JR, Garretson A, Dumont BL. Taxonomic assessment of two wild house mouse subspecies using whole-genome sequencing. Sci Rep. 2022;12(1):20866.
3. Kawakami M, Yamamura K. Cranial bone morphometric study among mouse strains. BMC Evol Biol. 2008;8:73.
4. Schwarz E, Schwarz HK. The wild and commensal stocks of the house mouse, *Mus musculus* Linnaeus. J Mammalogy. 1943;24(1):59-72.
5. Kuroda N. Korean mammals preserved in the collection of Marquis Yamashina. American Society of Mammalogists. 1934;15(3):229-39.
6. Ellerman JR, Morrison-Scott TCS. Checklist of Palearctic and Indian mammals 1758 to 1946. 2nd ed. London: British Museum (Natural History); 1951.
7. Mori T. On some small mammals from the Isl. Utsuryo, Chosen (II). Journal of Chosen Natural History Society. 1938;23:16-8.
8. Nam H, Kim YY, Kim B, Yoon WK, Kim HC, Suh JG. Genetic and morphometric characteristics of Korean wild mice (KWM/Hym) captured at Chuncheon, South Korea. Lab Anim Res. 2018;34(4):311-6.
9. Oh YS, Park JH, Lee YS. Morphogenetical survey of Korean wild mice. Korean J Lab Ani Sci. 1988;4(1):57-63.
10. Suh JG, Do SG, Kim CS, Seong JK, Hyun BH, Oh YS. Morphogenetical survey and distribution of the Fv4r gene in Korean wild mice. Korean J Lab Ani Sci. 1999;15(1):23-7.
11. Suh JG, Do SG, Kim CS, Lee SR, Oh SH, Kim HJ, et al. Morphogenetical survey of wild mice captured from Anmyon-up and Kumi city. Korean J Lab Ani Sci. 2000;16(2):107-113.
12. Myoshu H, Iwasa MA. Colonization and Differentiation Traits of the Japanese House Mouse, *Mus musculus* (Rodentia, Muridae), Inferred from Mitochondrial Haplotypes and External Body Characteristics. Zoolog Sci. 2018;35(3):222-32.
13. Iwasa MA, Udagawa M. Genetic and morphological characterizations of house mice on the Miura Peninsula, central Honshu, Japan. Mamm Stud. 2016;41:223-8.
14. Marshall JT. Identification and scientific names of Eurasian house mice and European allies, subgenus *Mus* (Rodentia: Muridae). Virginia: Privately published at Springfield; 1998.
